# Collecting Health and Exposure Data in Australian Olympic Combat Sports: Feasibility Study Utilizing an Electronic System

**DOI:** 10.2196/humanfactors.9541

**Published:** 2018-10-09

**Authors:** Sally Bromley, Michael Drew, Scott Talpey, Andrew McIntosh, Caroline Finch

**Affiliations:** 1 Physical Therapies Department Australian Institute of Sport Canberra Australia; 2 Australian Centre for Research into Injury in Sport and its Prevention Federation University Australia Ballarat Australia; 3 School of Health and Life Sciences Federation University Australia Ballarat Australia; 4 School of Health and Medical Sciences Edith Cowan University Perth Australia

**Keywords:** online, health, injury & prevention, athletic performance, internet, mobile phone

## Abstract

**Background:**

Electronic methods are increasingly being used to manage health-related data among sporting populations. Collection of such data permits the analysis of injury and illness trends, improves early detection of injuries and illnesses, collectively referred to as health problems, and provides evidence to inform prevention strategies. The Athlete Management System (AMS) has been employed across a range of sports to monitor health. Australian combat athletes train across the country without dedicated national medical or sports science teams to monitor and advocate for their health. Employing a Web-based system, such as the AMS, may provide an avenue to increase the visibility of health problems experienced by combat athletes and deliver key information to stakeholders detailing where prevention programs may be targeted.

**Objective:**

The objectives of this paper are to (1) report on the feasibility of utilizing the AMS to collect longitudinal injury and illness data of combat sports athletes and (2) describe the type, location, severity, and recurrence of injuries and illnesses that the cohort of athletes experience across a 12-week period.

**Methods:**

We invited 26 elite and developing athletes from 4 Olympic combat sports (boxing, judo, taekwondo, and wrestling) to participate in this study. Engagement with the AMS was measured, and collected health problems (injuries or illnesses) were coded using the Orchard Sports Injury Classification System (version 10.1) and International Classification of Primary Care (version 2).

**Results:**

Despite >160 contacts, athlete engagement with online tools was poor, with only 13% compliance across the 12-week period. No taekwondo or wrestling athletes were compliant. Despite low overall engagement, a large number of injuries or illness were recorded across 11 athletes who entered data—22 unique injuries, 8 unique illnesses, 30 recurrent injuries, and 2 recurrent illnesses. The most frequent injuries were to the knee in boxing (n=41) and thigh in judo (n=9). In this cohort, judo players experienced more severe, but less frequent, injuries than boxers, yet judo players sustained more illnesses than boxers. In 97.0% (126/130) of cases, athletes in this cohort continued to train irrespective of their health problems.

**Conclusions:**

Among athletes who reported injuries, many reported multiple conditions, indicating a need for health monitoring in Australian combat sports. A number of factors may have influenced engagement with the AMS, including access to the internet, the design of the system, coach views on the system, previous experiences with the system, and the existing culture within Australian combat sports. To increase engagement, there may be a requirement for sports staff to provide relevant feedback on data entered into the system. Until the barriers are addressed, it is not feasible to implement the system in its current form across a larger cohort of combat athletes.

## Introduction

Injury and illness can markedly impair an athlete’s performance, both in training and competition [1]. Injury and illness monitoring is the foundation stage of accepted prevention frameworks and can be described as the routine collection and reporting of injury and illness data [2,3]. Results from a recent review have indicated that there is a lack of high-quality, prospective injury and illness data published across the Olympic combat sports of judo, boxing, taekwondo, and wrestling [4]. Only one high-quality study was identified in the review, which was in the sport of judo [5]. In this study on judo, a dedicated medical team worked alongside coaches to prospectively collect, analyze, and act upon health-related information on a daily basis [5], thereby potentially enhancing the capture of injuries and illnesses. In Australia, combat sports organizations are limited in their ability to hire medical personnel to collect and report on injury and illness data. Therefore, there is a need to utilize online data systems, which can be accessed from across the country. With many athletes owning or having access to personal electronic devices, online systems have the potential to be easily administered to collect health [6] and training data directly from athletes [7].

A large portion of the epidemiological literature on combat sports details injuries and illnesses, which were sustained by athletes at competitions [8-17]. Collecting data solely at competition introduces the survival bias, whereby athletes who are severely injured and ill would be unlikely to be present at the competition where the data are being collected. Therefore, the injury and illness patterns described in these studies may not be accurate in relation to the overall athlete health. Monitoring athletes both in and out of competitions can address the survival bias; moreover, it can enhance the capture of recurrent health problems. Work from the Oslo Sports Trauma Research Centre shows that the weekly administration of injury and illness questionnaires is superior to the monthly administration for the capture of reoccurring injuries or illnesses [6]. With the increasing evidence that modified training due to injury and illness can also lead to a loss in long-term performance [18,19], it is important to give athletes the tools to self-report on their health and well-being.

An online system termed the Athlete Management System (AMS; Smartabase, Fusion Sport, Brisbane, Australia) has been adopted by the Australian Institute of Sport (AIS) to collect and store the health-related data of Australian high-performance athletes. The AMS allows the capture of recurrent health problems by providing athletes with an avenue to self-report injuries, illnesses, and training information. While not being designed specifically for each sport, the AMS meets the needs of a range of sports stakeholders, including doctors, physiotherapists, and coaches who can access, add to, and act on athlete health and training data. Training status, injury, and illness have been linked to performance outcomes in track and field by utilizing data collected via the AMS [19]. The AMS has also been utilized to promote shared decision making in volleyball around the risks and benefits of athletes participating in camps and competitions [1]. The data collection tools within the AMS can be customized to some degree; however, a limitation of the system is that the overall design remains the same regardless of the sport it is utilized for. In addition, the AMS does not assist with interpreting data once it is entered. To obtain information that can be fed back to coaches and athletes, a certain amount of work is required by sports personnel. In track and field, water polo, volleyball, and soccer, the sports staff who interpret and disseminate feedback based on the AMS data are physiotherapists and sports scientists based at the AIS. In a previously utilized cost-effective method, [5], team physiotherapists have collected data and provided feedback to coaches and athletes. In a recent study of 131 athletes across a range of sports, the provision of feedback was shown to enhance the uptake and engagement with an online self-report system [7]. Unlike Australian volleyball and track and field, there are no dedicated support staff, such as team physiotherapists, that drives monitoring and provides feedback for Australian combat sports programs. Due to a lack of support staff, it is unknown whether utilizing the AMS to monitor combat sports athletes and collect injury and illness data will be feasible.

The objectives of this paper are to (1) report on the feasibility of utilizing the AMS to collect longitudinal injury and illness data of combat sports athletes and (2) describe the type, location, severity, and recurrence of injuries and illnesses that an elite cohort of athletes experience across a 12-week period.

## Methods

### Participants

A feasibility study was implemented, and the source population was drawn from internationally competitive athletes in judo, boxing, taekwondo, and wrestling, who were affiliated with the AIS Combat Centre. Participants were recruited in April 2016, during an Olympic preparation camp. Of note, 5 eligible athletes were unable to attend the camp and were, therefore, contacted individually.

The inclusion criteria were elite and developing elite athletes who were affiliated with the AIS Combat Centre. Elite athletes were defined as those who had competed internationally for World Championship and Olympic qualification events in the previous 12 months. Developing elite athletes were defined as those who had competed internationally in Junior Grand Slams, Junior World Cups, and Junior World Championships in the previous 12 months. The exclusion criteria were athletes who only competed domestically and those who were not affiliated with the AIS Combat Centre. This project received ethical approval from an Australian Human Research Ethics Committee (approval number A16-023).

### Electronic Data Collection

In this study, we utilized 2 tools within the AMS: (1) a tool designed to capture training load and injuries for each training session termed “session monitoring” and (2) the Health Problems Questionnaire (HPQ) [6]. The AMS is accessible from personal computers, tablets, and phones and can be utilized both online and offline to record a range of training and health-related data. The session monitoring and HPQ tools were displayed on the AMS home screen, which was visible to athletes after logging in with their unique identification and password. The session monitoring and HPQ tools were specifically selected because they collected data on the training status and the degree to which injury and illness affected the training quality, respectively. Together, the tools allow athletes to report injuries and illnesses and whether they trained without modification and the degree to which they needed to modify their training because of injury and illness.

The session monitoring tool recorded information about the type, duration, and intensity of training sessions and whether athletes experienced any injuries. If the athletes answered “yes” to sustaining an injury, they were prompted to further document the affected area on an electronic body map and were asked to provide additional written detail about the injury. The training load was computed as the rating of perceived exertion multiplied by the session duration for each training session. This is a cost-effective method, previously utilized in judo to quantify the training load [20-24]. Additionally, rapid shifts in training load have been associated with injury incidence and severity, and they represent a method of calculating the exposure [25].

**Figure 1:**
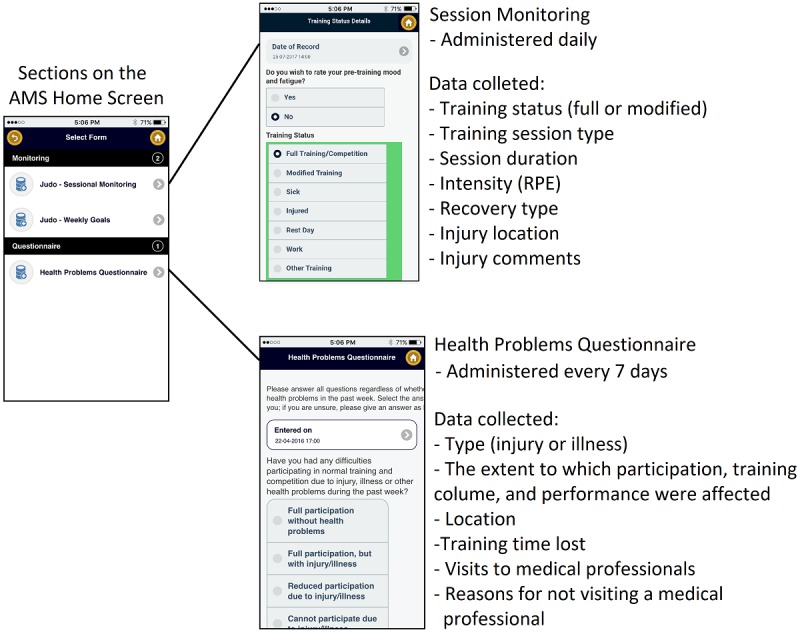
Electronic data collection tools accessible from the Athlete Management System (AMS) home screen and the frequency of administration. RPE: rating of perceived exertion.

The HPQ is a questionnaire designed to capture athlete self-reported injuries and illnesses, which may or may not result in lost training time [6] and is embedded within the AMS. During the study, when an athlete clicked on the HPQ section within the AMS, 4 questions appeared related to the degree to which the athlete experienced a health problem that week. If they answered that a health problem had affected them, additional questions appeared that requested more detail about that health problem. The HPQ allows athletes to report on up to 10 health problems each week by asking “Have you experienced any other health problems this week?” as the final question. If the athletes answer yes, they are taken back to the start of the questionnaire. Previous literature utilizing this questionnaire found that a cohort of 142 Olympic athletes collectively documented 15 health problems per week; therefore, the option to report 10 health problems per athlete per week was determined to be sufficient [6]. The severity of combined health problems (injuries and illnesses) was calculated by scoring the responses to the 4 key questions from 0 (no problems) to 25 (maximum level), as has been published previously [26]. Where athletes reported the same injury and illness across both the session monitoring and HPQ tools, the HPQ data was omitted for that week to avoid duplication. Figure 1 displays the data captured across each tool and the frequency of administration.

Upon enrollment, the principal researcher (SB) tested each athlete’s access to the electronic system by accessing the AMS app on a smartphone and logging in as each athlete. Access to each tool was checked for each athlete; however, no data were saved. Study information was presented by SB as part of an introductory session of the Olympic preparation camp, where athletes performed administrative tasks and were briefed on the camp schedule. Upon enrollment in the study, written informed consent to contact the athlete’s treating health professionals (medical practitioners, physiotherapists, etc) and their coaches was obtained in case there was a need to verify any entered data. In addition, consent was obtained for researchers to be able to contact the participant with reminders (eg, phone, email, face-to-face) to enter their data. After the camp, detailed instructions of how to access the AMS and enter data in both session monitoring and HPQ sections were emailed to enrolled athletes. Athletes were free to withdraw their consent at any time without penalty. Reminders and requests were sent to athletes when data were missing or incomplete. Sample communications are presented in Multimedia Appendix 1. Coaches were not utilized as a means to increase the athlete engagement with the AMS; this decision was made so that a coach’s previous experience with the system, if any, would not affect this study.

### Data Analysis

**Figure 2:**
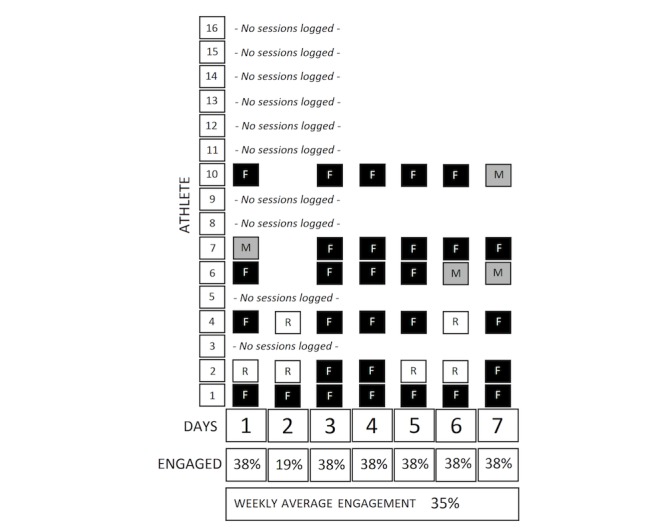
Method of calculation for weekly compliance rates. R: rest day; F: full training; M: modified training. Gaps indicate no data were entered for that day by that athlete.

Daily engagement with the session monitoring section of the electronic system was calculated and expressed as a weekly average. For each day of the study period, the number of athletes who made a session monitoring entry (which included an option for rest days) was divided by the total number of athletes enrolled in the study. This daily result was then averaged across 7 days to give a weekly cohort engagement score, expressed as a percentage (Figure 2). The weekly cohort engagement score indicates an athlete’s autonomy to self-engage with the AMS.

Descriptive statistics were used to determine the level of uptake (percentage of athletes who were engaged within the first week of data collection) and engagement across the combat sports. In addition, injuries and illnesses were coded using the Orchard Sports Injury Classification System (OSICS) version 10.1 and the International Classification of Primary Care, version 2 (ICPC-2) [27,28]. Days lost to injury and illness were recorded, and the severity of injuries or illnesses were calculated using published methods [26]. Data were analyzed using Stata (13 IC, Stata Corp, College Station, TX, USA).

## Results

### Uptake and Engagement With the Electronic System

In total, 21 athletes attended the Olympic preparation camp (boxing: 5 [3 females, 2 males], judo: 9 [4 females, 5 males], taekwondo: 3 [2 females, 1 male], wrestling: 4 [4 males]), and an additional 5 who did not attend the camp were contacted (boxing: 3 [1 female, 2 males], judo: 1 [1 male], taekwondo: 1 [1 male]), totaling 26 athletes (10 females, 16 males). Of the 26 athletes, 9 judo (4 females, 5 males) and 7 boxing (4 females, 3 males) athletes were enrolled in this study (response rate, 55%), with no taekwondo or wrestling athletes being enrolled. Of all the registered participants, 13% (2/16) participants entered data across the entire study period, 56% (9/16) entered data intermittently, and 31% (5/16) did not enter any data (boxing: 1 [1male], judo: 4 [3 females, 1 male]). Data collection ranged from 84 to 109 days, equaling 12-15 weeks, depending on where the athletes were recruited within the recruitment period. Including the recruitment period, there was the potential to administer 224 weekly HPQs; however, only 27.2% (61/224) HPQs were completed. During the study, there was potential to collect 1744 days of data, yet only 34.6% (603/1744) days were logged into the online system. Table 1 summarizes the athlete characteristics and engagement rates across the monitoring period.

**Table 1:** Participant characteristics and engagement rates for the study period.

Sport	Competitive status	Engagement (days recorded), n (%)	Health Problems Questionnaire engagement (weeks recorded), n (%)
Judo	Developing elite	61 (75.3)^a^	8 (80)^a^
Judo	Developing elite	104 (95.4)	9 (64)
Judo	Elite	71 (65.1)	9 (64)
Judo	Elite	61 (56.5)	6 (43)
Judo	Elite	50 (45.9)	0 (0)
Boxing	Elite	82 (75.2)	13 (93)
Boxing	Developing elite	54 (50.0)	9 (64)
Boxing	Elite	32 (41.6)^a^	5 (50)ᵃ
Boxing	Elite	12 (11.0)	3 (21)
Boxing	Elite	9 (8.3)	1 (7)
Boxing	Elite	69 (87.3)^a^	0 (0)^a^

^a^Four weeks into the study, 3 athletes joined, 1 “developing elite” and 2 “elite”; therefore, engagement for these 3 athletes was measured on the basis of 81, 77, and 79 days, respectively, and 10 HPQs.

**Figure 3:**
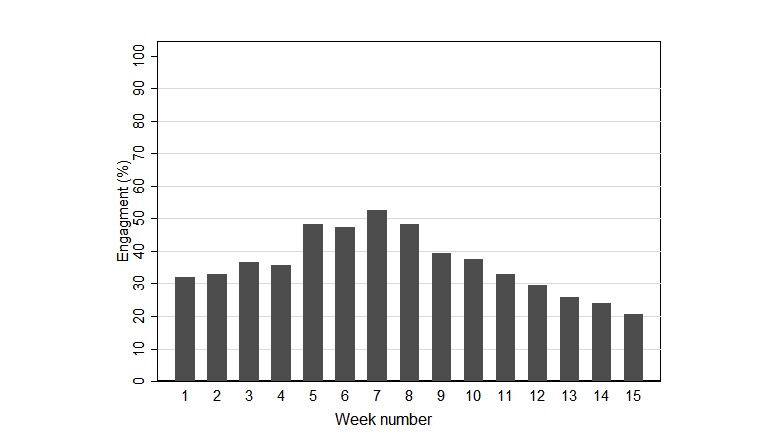
Average engagement rate across the cohort during each week of the study period.

The level of athlete engagement with the session monitoring section of the electronic system began at 32%, increased to 53% at week 7, and slowly declined to 21% at week 15. Figure 3 depicts the level of engagement across the study period.

Over the study period, 161 separate communications were made by the principal researcher to the participating athletes via short message service (SMS) text messages (81/161, 50.3%), email (38/161, 23.6%), phone calls (2/161, 1.2%), face-to-face conversations (14/161, 8.6%), and a combination of methods (26/161, 16.1% SMS text messages plus email). The estimated time commitment for the principal researcher (SB) was 90 seconds per communication, equal to approximately 16 minutes per week of reminders and troubleshooting. In addition, SB had face-to-face conversations with 8 coaches of the enrolled athletes to reinforce the study benefits and made 17 communications to athletes who did not attend the camp to encourage them to engage with the tools (Multimedia Appendix 1).

### Injuries and Illnesses

Over the study period, 23 unique injury codes and 7 unique illness codes were captured. There were 93 repeats of injury codes and 7 repeats of illness codes across both the tools, totaling 130 injury and illness incidents. Table 2 outlines the body area and prevalence of injuries and illnesses experienced by combat athletes across the study period.

**Figure 4:**
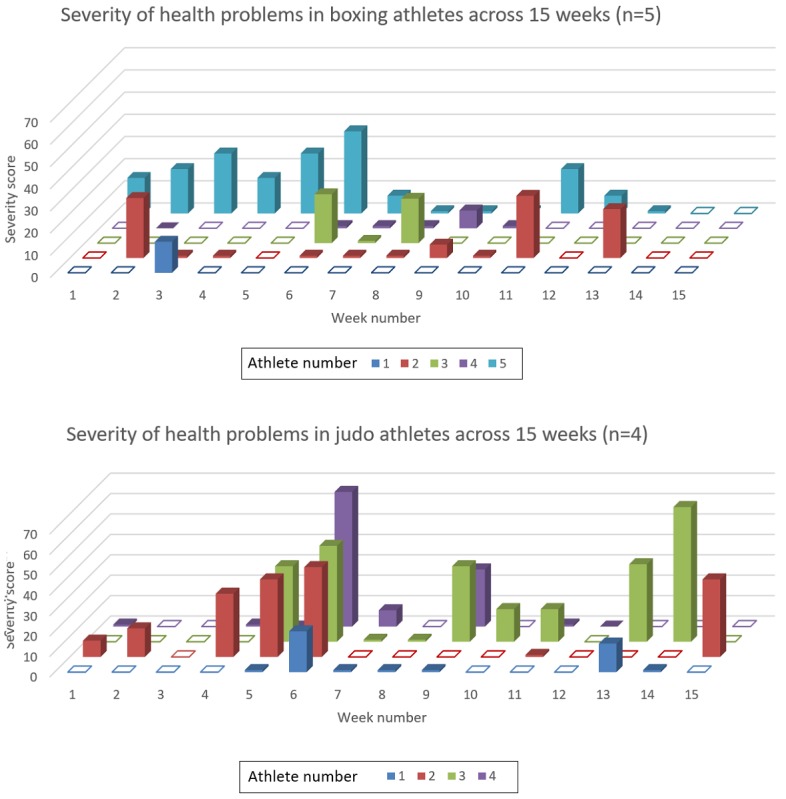
Severity of health problems in boxing (n=5) and judo (n=4) athletes across the study period (unfilled squares: uncompliant; shaded squares: compliant but with no reported health problems).

Of note, 2 injuries affected one particular athlete for 8 weeks each, often being logged in the same session. In addition, 4 judo and 5 boxing (9/16, 56%) athletes completed HPQs throughout the monitoring period; however, their session monitoring entries were mostly inconsistent. Figure 2 displays the severity of health problems experienced by these athletes for each week of the study period. A taller column for an athlete in a given week indicates that a health problem affected their training to a greater degree. Where there is no column for athletes, they either did not complete an HPQ or experienced no health problems that affected their training. In general, the combined severity of health problems (injuries and illnesses) captured suggests that, in this specific cohort, judo athletes tended to report more severe health problems than the boxers (Figure 4).

### Time Lost to Injury and Illness

In this study, 2 injuries and 3 illnesses in 3 athletes (5/30, 16%, of unique injury and illness codes) resulted in lost training time. Time-loss for these events did not exceed 2 days. Generally, athletes trained through injury and illness for all remaining injuries and illnesses.

**Table 2:** Injuries and illnesses experienced by combat sports athletes (N=16) across the study period according to the sport and the complaint and area.

Complaint and area		Judo, n	Boxing, n	Total, n
**Illness^a^**				
	Abdominal pain or general cramps	1	N/A^b^	1
	Chest infection	N/A	N/A	N/A
	Chest symptom or complaint	2	1	3
	Fever	1	N/A	1
	General symptom or other complaint	4	1	5
	Lymph gland(s) enlarged or painful	2	1	3
**Injury^c^**				
	Foot	6	N/A	6
	Head	2	1	3
	Hip and groin	2	1	3
	Knee	2	41	43
	Lower leg	N/A	3	3
	Lumbar spine	3	2	5
	Nerve issue, arm	N/A	N/A	N/A
	Shoulder	2	7	9
	Thigh	9	1	10
	Trunk and abdomen	4	4	8
	Wrist and hand	8	19	27

^a^Total illness: judo 10 (8%), boxing 3 (2%); percentages are calculated based on the total number of illnesses collected during the monitoring period.

^b^N/A: not applicable.

^c^Total injury: judo 38 (29.2%), boxing 79 (60.8%); percentages are calculated based on the total number of injuries collected during the monitoring period.

## Discussion

### Principal Findings

This study reports the feasibility of utilizing the AMS to collect injury and illness data of combat sports athletes over a 12-week period and provides information on the injuries and illnesses sustained by the cohort during this time. A key finding is that engagement with the AMS was low; therefore, strategies to increase engagement will need to be specifically addressed if the AMS is to be fully implemented as a monitoring tool across the combat sports. However, data collected via the system illustrates a need for monitoring as the cohort experienced multiple health problems that tended to recur or progress toward chronicity.

### Engagement With the Athlete Management System

Coach endorsement is one of the most important socioenvironmental factors for promoting the initial uptake of a monitoring system [7]. In an attempt to increase coach endorsement and subsequent athlete engagement, this project was launched at an Olympic preparation camp where coaches were directly informed about the study benefits. Despite launching at a high-profile camp, athlete engagement with the AMS was low across the study period, and at its peak, only half of the athletes were entering data; this aligns with a recently published work investigating the uptake of a similar self-report system in judo, swimming, and volleyball (50%, 61%, and 56%, respectively) [7]. A number of factors potentially influenced engagement with the AMS, including access to the internet, the design of the system, coach views on the system, previous experiences with the system, and the existing culture within Australian combat sports.

Most athletes undertook competition travel during the monitoring period where a number of issues could have limited their intent to engage with the AMS. An offline mode is available at the log-in screen; however, once an athlete is logged in, the offline feature is less obvious. An athlete using the system in Australia may not log out when overseas; therefore, he or she may forget that this feature is available. Overall, the engagement rates indicate that the system is not intuitive and requires additional motivation and effort to use. Athletes who engaged with the system in the first week of the study were more likely to continue to engage thereafter. Internal motivation likely came from the study being launched at an Olympic preparation camp, where there may have been social desirability to use the system. Utilizing the preparation camp to increase internal motivation was the intent, as higher internal motivation has been linked to higher engagement with monitoring tools [7].

In contrast, those who did not engage early in the study did not change their behavior, even when contacted by the research team and encouraged to utilize the system. This could be attributed to a lack of additional encouragement from coaches to utilize the system as some had used the system before and were not convinced of its benefit. The AMS had been previously implemented in boxing across a small cohort, and monitoring and engagement was driven by a single staff member employed by Boxing Australia Limited. This staff member applied penalties for failing to engage with the system, rather than highlighting the benefits of such a system to athletes and coaches. It is possible that in this early trial, some coaches and athletes had good experiences (did not receive punishments) and some had bad experiences (received penalties) and that these previous experiences affected their intent to engage in the study. To avoid the influence of a coach’s previous experience, athletes were contacted directly and coaches were not utilized to increase engagement. However, it is possible that some coaches may have expressed their opinion on the system to athletes at some point during the study.

As mentioned above, the outputs of the AMS require a level of interpretation, and therefore, feedback to athletes on their entered data is not immediate. This is a significant failing and is likely to contribute to the low engagement rates. The provision of immediate and relevant feedback to athletes has been cited as one of the key determinants as to whether an athlete will engage with a self-report tool [29]. In addition, feedback must be from a reputable and relevant source, such as a coach or sports staff member who works closely with athletes participating in monitoring programs [30]. Regular contact from the research team did not influence the rate of entry, whether used as a reminder or as positive reinforcement; therefore, it is possible that athletes did not view the source of feedback as relevant or reputable. Overall, approximately one-third of training days and one-quarter of HPQs were collected across the study period, indicating that it is not currently feasible to utilize this system to report injury and illness under the current combat sports structure. A primary difference between studies that have successfully collected high-quality data through the AMS and this study is that support staff were employed by those other national sports organizations to interpret and provide relevant feedback on entered data to coaches and athletes. In combat sports, no staff are currently employed to provide such services. If the AMS is to be fully implemented, it will likely require dedicated staff to maximize engagement and subsequent data quality.

### Injuries and Illnesses Within the Cohort

Despite low engagement with the monitoring system, a large number of health problems were reported through it, the majority of which did not affect training time. Of 603 recorded training and competition days, only 7 days were lost and 11 days modified due to injury and illness. There was double the number of repeated injury codes than unique injury codes, suggesting that athletes carried chronic injuries or injuries had a high recurrence. Data collected via online systems in Paralympic athletes showed that, on average, athletes sustained 0.31 new injuries per week (15 injuries recorded by 12 athletes over 4 weeks) [31]. The Paralympic study utilized similar injury and illness definitions to those in this study, which allowed the capture of injuries that did not result in lost training or competition time but affected the quality of training or competition. In this study, combat athletes reported more than double this amount—116 injuries over 12 weeks, equaling 0.88 injuries reported per week. Combat athletes were able to continue training irrespective of injury and illness events in 97% of cases. Together, these results suggest that this cohort of combat athletes maintained their training despite experiencing repeated health issues. In the cohort, the areas that had the highest injury frequency were the thigh in judo (n=9) and knee in boxing (n=41), with wrist and hand injuries being second highest in both sports (n=8 and n=19, respectively). This is in contrast with previous combat sports research, which indicates that the head or face is the most injured area in boxing training [32,33] and that the lower back is the most injured area in judo training [5]. This difference could be attributed to the injury and illness definitions utilized in previous studies, which have focused on injuries and illnesses that resulted in medical treatment and lost training time. This is a noted limitation in the combat sports literature [4] and does not account for injuries that may be self-managed by athletes, as discussed below.

### Considerations for Monitoring Systems in Combat Sport

Self-report systems allow athletes to report self-managed health problems, which may not be apparent during training or require an urgent visit to a medical professional. In previous combat sports studies, data have been collected using paper-based systems and face-to-face consultations between medical staff, coaches, and athletes [5,32]; this leaves a gap in the collection of self-reported injuries and limits the ability to make comparisons between rates of self-managed health problems and those which require treatment by medical practitioners. A strength of this study is that the session monitoring tool within the AMS allowed athletes to reflect on a single training session; this likely increased the capture of these self-managed issues, which appear to have little impact on training time yet appear to impact performance during training. Additionally, in previous judo and boxing research, all health problems have been treated in separation and, therefore, smaller problems may not have been recorded. Subsequently, the relationships between small and large injuries in combat sports could not be investigated as they have been in other sports [34]. Due to sample size limitations and low engagement, an analysis of the relationships between injuries is not possible; however, this study shows that using the HPQ and session monitoring tools within the AMS, these health problems can be documented.

In this study, athletes reported that they were able to train through the majority of their health problems; however, these problems led to reductions in performance, pain, or modified training and often lasted multiple weeks. This result may have been overlooked if the definition of injury had been restricted to lost training time, rather than relating to physical complaints. Only 4% of health problems would have been captured if a “time-loss” definition had been used in this cohort, meaning that 97 reports of health problems affecting athletes would not have been included in the final pool of injury and illness data. The majority of these (63 health problems) were repeats of previous OSICS or ICPC-2 codes, indicating that the issues were more recurrent than acute in nature. The phenomenon of training through injury may be unique to this particular cohort; however, due to combat sports being contact in nature with the goal to physically dominate an opponent, it is likely that training while carrying an injury is part of combat sports culture. Therefore, utilizing only missed training or competition time to define combat athlete injury and illness may not allow a full capture of injuries or illnesses in these athletes. To improve outcomes for athletes, health problems that affect both training time and the quality of health should be considered when identifying where prevention programs are targeted.

### Limitations and Considerations for Future Research

Results from this study provide preliminary data detailing injuries or illnesses in this cohort of Australian judo and boxing athletes. Generalizing the injury and illness results of our study to the wider combat sports population is not appropriate due to the select cohort and the low engagement with the monitoring system. Furthermore, inconsistent engagement, both among athletes and across the monitoring period, likely affected the results. While these issues prevent application to the larger community of combat athletes, the study delivers important learnings around the utilization of the AMS as a monitoring system for combat sports. Reportedly, injury and illness monitoring allows the identification of injury and illness patterns and provides information for the development of intervention programs [2]. Despite the potential of AMS tools to collect high-quality data, a widespread implementation of the system in its current form is not feasible in Australian combat sports due to low engagement. Furthermore, issues with engagement could potentially be addressed by investing in the relevant medical or sports staff to assist with data interpretation and provision of timely feedback to athletes.

### Conclusions

Australian combat athletes appear to experience repeated health problems, yet there are no permanent processes in place to monitor the health of these athletes. Results from this study indicate that engagement with data reporting systems such as the AMS is poor, possibly due to system designs that fail to provide immediate and relevant feedback on entered data. To address these barriers, relevant staff who can provide feedback to coaches and athletes and troubleshoot problems are required. Until the barriers are addressed, it is not feasible to implement the system across a larger cohort of combat athletes.

## Appendix

Multimedia Appendix 1 Communication channel and sample messages of contact to participants.

## Abbreviations

AIS: Australian Institute of Sport
AMS: Athlete Management System
HPQ: Health Problems Questionnaire
ICPC-2: International Classification of Primary Care, version 2
OSICS: Orchard Sports Injury Classification System
SMS: short message service
