# Supplementary material for: Collecting Health and Exposure Data in Australian Olympic Combat Sports: Feasibility Study Utilizing an Electronic System
Source: JMIR Hum Factors. 2018 Oct 9;5(4):e27. doi: 10.2196/humanfactors.9541 (PMC6231822; doi:10.2196/humanfactors.9541)
Supplement: Multimedia Appendix 1 [file humanfactors_v5i4e27_app1.pdf]

Appendix 1: Communication channel and sample messages of contact to participants.

Communication channel	*Purpose*	*Message*
Email	Follow up after recruitment	Hi (name),<br>Thanks again for being part of my study on injury and illness in combat sport.<br>As part of the study, you are requested to complete a "Health Problems Questionnaire" each week on a Sunday. This questionnaire is accessible from the AMS home screen and some screen shots are attached for both iPhone and Android which show how to access the questionnaire.<br>We are aiming to capture once-off and recurring injuries and illnesses so if you have a persistent injury or illness problem, please enter it into the questionnaire for as many weeks as you have the problem. The questionnaire will take between 5-10minutes.<br>If you have any questions or problems please let me know,<br>(researcher contact details here)
Email and SMS via the AMS	Participant engaged	Nice work filling in the Health Problems questionnaire last week (athlete)! Using the results from this study we'll be able to see which injuries can be prevented in (sport), keeping you in the ring for longer! It will make a huge impact if you're able to fill this questionnaire in each week on the same day, so that means your next one is this Friday. Thanks again, (researcher).<br>(researcher contact details here)
SMS	Participant reminder	Hi (athlete), just a reminder that your health problems questionnaire is due today :)
SMS	Participant reminder	Hi (athlete), great work filling in your training log! If you could fill in a health problems questionnaire this week that would be great. Let me know if you need any assistance :)
Email and SMS via the AMS	Lost participant engagement	Hi (athlete),<br>It's been a few days since you've logged any training into the AMS. If you take rest days it's also useful to log them so that we can provide you accurate feedback. If you're having any troubles with the AMS please let me know as I am happy to assist, (researcher).<br>(researcher contact details here)
Email and SMS via the AMS	Well done message	Hi (athlete),<br>We just want to say great work filling in your training log on the AMS! Thanks for being so consistent, (researcher).<br>If you ever have any problems with your AMS system please feel free to contact me directly, (researcher contact details here).

Footnote: AMS, Athlete Management System; SMS, Short Message Service.
